# Machine learning-based integration identifies the ferroptosis hub genes in nonalcoholic steatohepatitis

**DOI:** 10.1186/s12944-023-01988-9

**Published:** 2024-01-23

**Authors:** Longfei Dai, Wenkang Yuan, Renao Jiang, Zhicheng Zhan, Liangliang Zhang, Xinjian Xu, Yuyang Qian, Wenqi Yang, Zhen Zhang

**Affiliations:** Department of General Surgery, The First Affiliated Hospital of Anhui Medical University, 218 Jixi Road, Hefei, 230022 Anhui Province China

**Keywords:** Machine learning, Ferroptosis, NASH, ZFP36, Diagnosis

## Abstract

**Background:**

Ferroptosis, is characterized by lipid peroxidation of fatty acids in the presence of iron ions, which leads to cell apoptosis. This leads to the disruption of metabolic pathways, ultimately resulting in liver dysfunction. Although ferroptosis is linked to nonalcoholic steatohepatitis (NASH), understanding the key ferroptosis-related genes (FRGs) involved in NASH remains incomplete. NASH may be targeted therapeutically by identifying the genes responsible for ferroptosis.

**Methods:**

To identify ferroptosis-related genes and develop a ferroptosis-related signature (FeRS), 113 machine-learning algorithm combinations were used.

**Results:**

The FeRS constructed using the Generalized Linear Model Boosting algorithm and Gradient Boosting Machine algorithms exhibited the best prediction performance for NASH. Eight FRGs, with ZFP36 identified by the algorithms as the most crucial, were incorporated into in FeRS. ZFP36 is significantly enriched in various immune cell types and exhibits significant positive correlations with most immune signatures.

**Conclusion:**

ZFP36 is a key FRG involved in NASH pathogenesis.

**Supplementary Information:**

The online version contains supplementary material available at 10.1186/s12944-023-01988-9.

## Introduction

There is mounting evidence implicating non-alcoholic fatty liver disease (NAFLD) result in persistent liver damage [1, 2]. The increasing incidence of NAFLD affects human survival time [3]. In the early stages, NAFLD manifests as nonalcoholic fatty liver disease [4]; nevertheless, advanced-stage NAFLD is characterized by necroinflammatory responses and increased hepatic fat accumulation. NASH can result from the accumulation of hepatic fat and the infiltration of inflammatory cytokines [5]. NASH liver cells can develop fibrosis, hardening, and even cancer if no intervention is performed [6]; thus, early identification of NASH is crucial. NASH is traditionally diagnosed using a liver biopsy [7], but its invasive nature limits its clinical application. Another challenge in the management of NASH is the lack of effective treatments. Although lifestyle modifications and increased physical activity are considered primary therapeutic measures for NASH, research suggests that their outcomes are suboptimal [8]. Thus, the search for therapeutic targets in NASH remains a critical endeavor.

Ferroptosis entails the activation of reactive oxygen species (ROS) and lipid peroxidation by iron ions [9]. Ferroptosis likely contributes to the development of NASH because of the significant role of the liver in storing iron ions and regulating lipid metabolism [10]. NASH treatment may be enhanced by understanding the core ferroptosis genes in patients with NASH.

In machine learning, predictive models are constructed by identifying complex patterns in datasets or by correlating data with predictions [11]. This analytical discipline is useful for the development of optimal predictive models for disease diagnosis. Machine learning has been shown to provide robust risk models within clinical datasets, enabling the delineation of distinct patient cohorts based on clinical data [12, 13].

This study hypothesises that the innovative integration of multiple machine learning algorithms to identify FRGs would develop a non-invasive diagnostic model to better serve patients with NASH.

## Methods

### Obtaining GEO data

Seven cohorts containing information from NASH patients (GSE130970, GSE89632, GSE61260, GSE126848, GSE48452, GSE164760, and GSE63067) were selected from the GEO database (https://www.ncbi.nlm.nih.gov/geo/). Gene matrices were calculated using the platform files associated with each GEO dataset. The following table presents the platform file information for the seven GEO datasets. A merged dataset was created by merging the GSE130970 and GSE89632 datasets, after removing batch effects. The remaining five datasets were considered the external validation sets. Additionally, to increase the number of external validation sets, two additional validation cohorts (all-testing set cohorts and sample cohorts) were created (Supplementary Table 1).

### Filtering differentially expressed genes (DEGs) associated with Ferroptosis

The DEGs between the two groups were screened based on |logFC| > 0.585 and corrected *P*-values < 0.05. Additionally, 484 FRGs are available in the FerrDb V2 database (http://www.zhounan.org/ferrdb/current/) [14]. These genes were combined to identify 16 DEGs. The comprehensive bioinformatics database Metascape (https://metascape.org/gp/index.html#/main/step1) exhibits several enrichment analysis tools and resources [15]. The Metascape database was used to determine possible biological pathways and functional categories associated with ferroptosis-related DEGs.

### Constructing a ferroptosis-related signature (FeRS)

Twelve machine learning algorithms were selected for this study for the binary classification variables. The Enet, Ridge, Stepglm, and Least Absolute Shrinkage and Selection Operator (LASSO) algorithms are typically applied for regression problems, particularly when there are many features, to select the most important features and reduce the risk of overfitting. The Support Vector Machine (SVM), Linear Discriminant Analysis (LDA), glmBoost, Partial Least Squares Regression Generalized Linear Model (plsRglm), Random Forest, GBM, extreme Gradient Boosting (XGBoost), and Naive Bayes are all suitable for classification tasks in this study. A total of 113 algorithms were developed from the 12 machine learning algorithms. Two measures are implemented to mitigate the risk of overfitting. A cross-validation approach was used in the first step, with one algorithm for variable selection and the other for constructing the classification prediction model for each combination algorithm. Another approach involves increasing the number of validation datasets.

The areas under the receiver operating characteristic (ROC) curves were evaluated for the 113 machine-learning algorithms in eight cohorts. A heat map was used to show the results of the model. The best algorithm was selected from among 113 algorithms. Model genes filtered using the best algorithm were used to calculate the ROC values for diagnosing NASH in the eight cohorts.

### Pathway enrichment and immune infiltration analysis

GSVA analysis identified significantly enriched biological pathways [16]. Additionally, it was used to explore enriched biological pathways when the model genes were upregulated or downregulated. Differences in the enrichment of immune features and immune cells were investigated in model genes.

GENEMANIA was applied to identify genes that interact with the model genes [17]. Friendship analysis is a network topology-based analytical method used to explore interactions and relationships between genes or proteins in biological processes [18]. It relies on semantic similarity measurements based on GO, which means that associations are analyzed using the functional information of genes. Friendship analysis can help select the most significant genes from a large set of DEGs. Therefore, it was employed to evaluate the most crucial genes among the model genes.

### Single-cell analysis

The GSE159977 and GSE129516 datasets were downloaded. The former was separated into two groups, GSE159977(1) and GSE159977(2), each containing five human NASH samples. GSE129516 consists of three NASH mouse samples. To analyze the GSE159977(1), GSE159977(2), and GSE129516 datasets, the “Seurat” package was applied separately. First, the “PercentageFeatureSet()” function and all gene sets starting with MT- for mitochondrial quality control analysis were used to assess the proportion of mitochondrial genes in each cell to identify and exclude low-quality cells. To determine which features within the dataset displayed a high level of inter-cell variability, gene expression values were normalized using the “LogNormalize” method. For each dataset, 1500 features were selected for the PCA dimensionality reduction analysis, and the “JackStrawPlot()” and “ElbowPlot()” functions were used to determine the dimensionality of the dataset. Subsequently, cell clustering was performed, and the results were visualized using either the t-distributed stochastic neighborhood embedding method or the uniform surface approximation and projection method. Finally, the “SingleR” package was used to annotate different clusters as distinct immune cells. Additionally, differences in the expression of model genes among different immune cells were determined.

### Regulation factors of hub genes

The NetworkAnalyst platform was used in this investigation (https://www.networkanalyst.ca/) [19]. The JASPAR database was used to elucidate the regulatory network; the JASPAR database was selected and examined [20]. Furthermore, miRTarBase v8.0, was used to investigate miRNAs associated with the model genes [21]. Drug-targeting model genes were analyzed using the Drug-Gene Interaction database (DGIdb; http://dgidb.org/) [22]. The data were visualized using the Cytoscape software suite [23].

### Quantitative real-time polymerase chain reaction (qPCR)

Eleven pairs of normal and NASH liver tissues (NASH score > 5) were collected from the hospital, and qPCR was performed. The RNA was extracted from the samples and then reverse-transcribed into cDNA. Relative expression of the model genes was standardized with GAPDH and evaluated using the 2-ΔΔCt method. Box plots were generated with the GraphPad Prism software (version 9.0.0). Primers and reagents used are showed in Supplementary Table 2.

### Statistical analysis

**Fig. 1:**
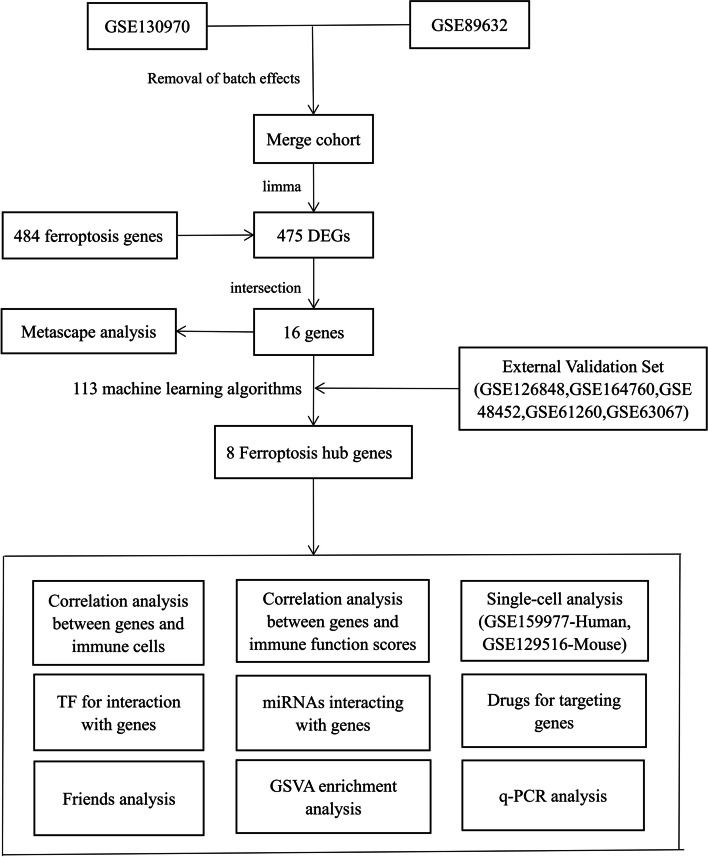
The flow chart of the research

The Mann-Whitney U-test was applied to compare the two subgroups that did not satisfy the t-test. Statistical significance was set at *P* < 0.05. 3. The concept of the full text is presented in Fig. 1.

## Results

### Cohort integration

**Fig. 2:**
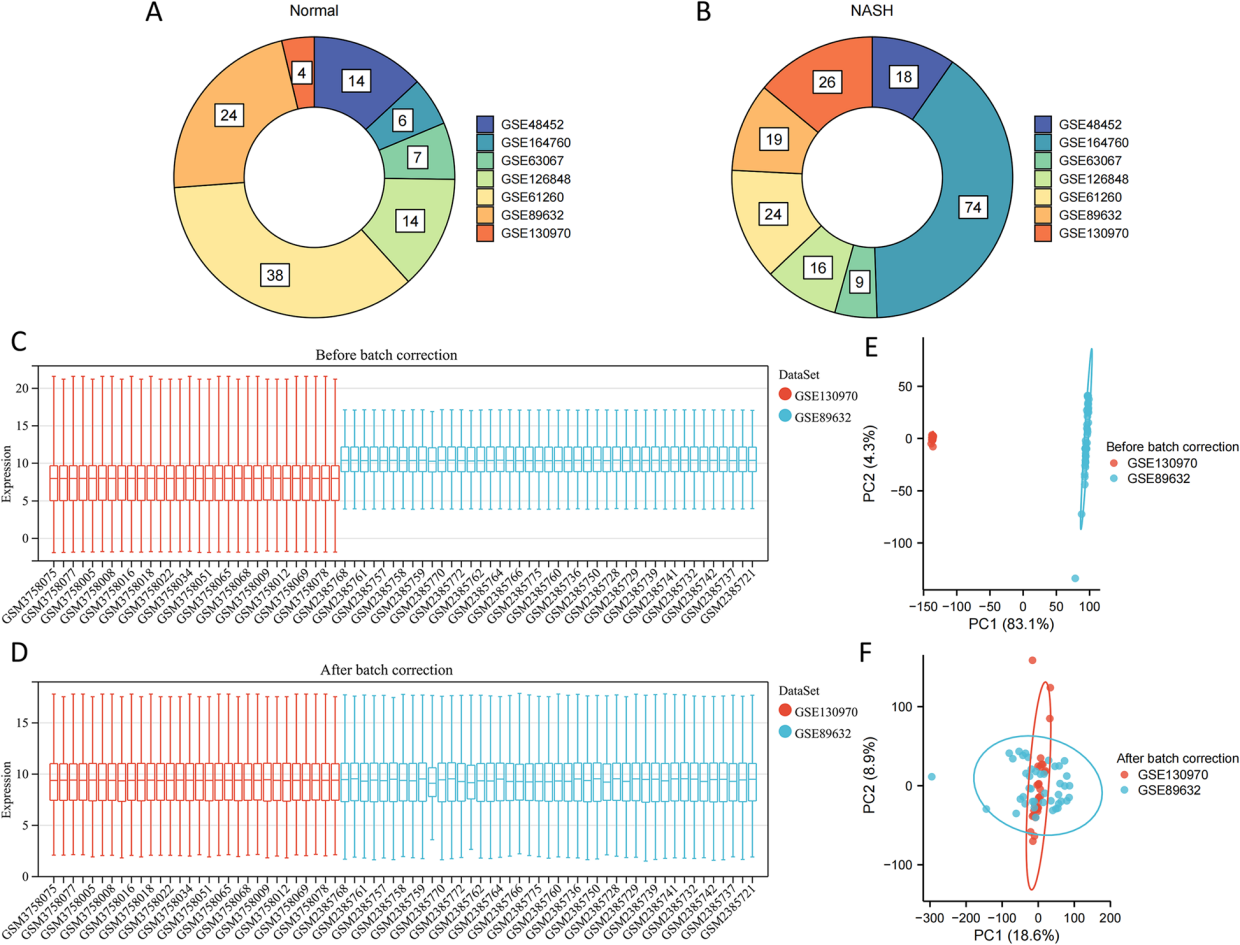
The process of data integration. **A-B** Statistics on the number of samples. **C, D** Before and after sample integration. **E-F** Principal component analysis (PCA) plots before and after the removal of the batch effect

Healthy and NASH samples from seven datasets were analyzed (Fig. 2A,B). After removing the batch effect, GSE130970 and GSE89632 were combined to create a training set (Fig. 2C-F).

### Sixteen ferroptosis-related DEGs associated with metabolism

**Fig. 3:**
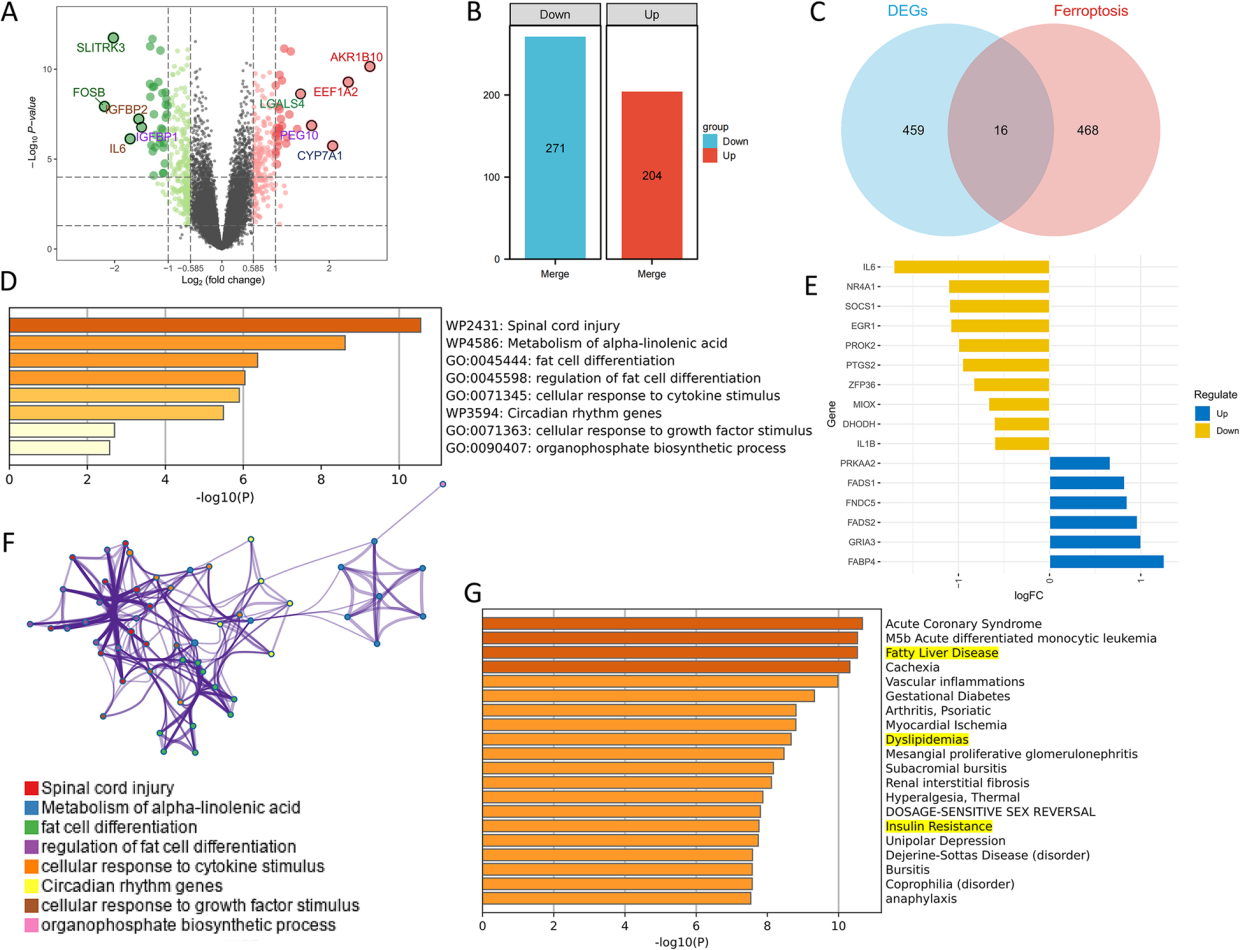
It was determined that 16 genes were involved. (**A**) Across the two groups, 475 differential genes (DEGs) were found. (**B**) Expression landscape of DEGs. (**C**) Sixteen common genes were recognized. (**D**) Expression patterns of the genes. (**E, F**) Biological pathway enrichment analysis. (**G**) Disease enrichment analysis

Four hundred seventy five DEGs were observed. A total of 271 genes were highly expressed in NASH, whereas the opposite was true for 204 genes (Fig. 3A, B). Sixteen ferroptosis-related DEGs were identified when these 475 DEGs intersected with 484 FRGs (Fig. 3C). Notably, in the NASH group, six genes were significantly upregulated, while 10 genes were significantly opposite (Fig. 3D).

These 16 genes were significantly enriched in pathways associated with “alpha-linolenic acid metabolism,” “adipocyte differentiation,” “regulation of adipocyte differentiation,” and “circadian rhythm” (Fig. 3E, F). Consequently, the metabolic pathways are intertwined with NASH development in a complex manner. There was also a significant association between these genes and the etiologies of NASH, such as “fatty liver disease,” “lipid metabolism abnormalities,” and “insulin resistance” (Fig. 3G).

### Development of an FeRS

**Fig. 4:**
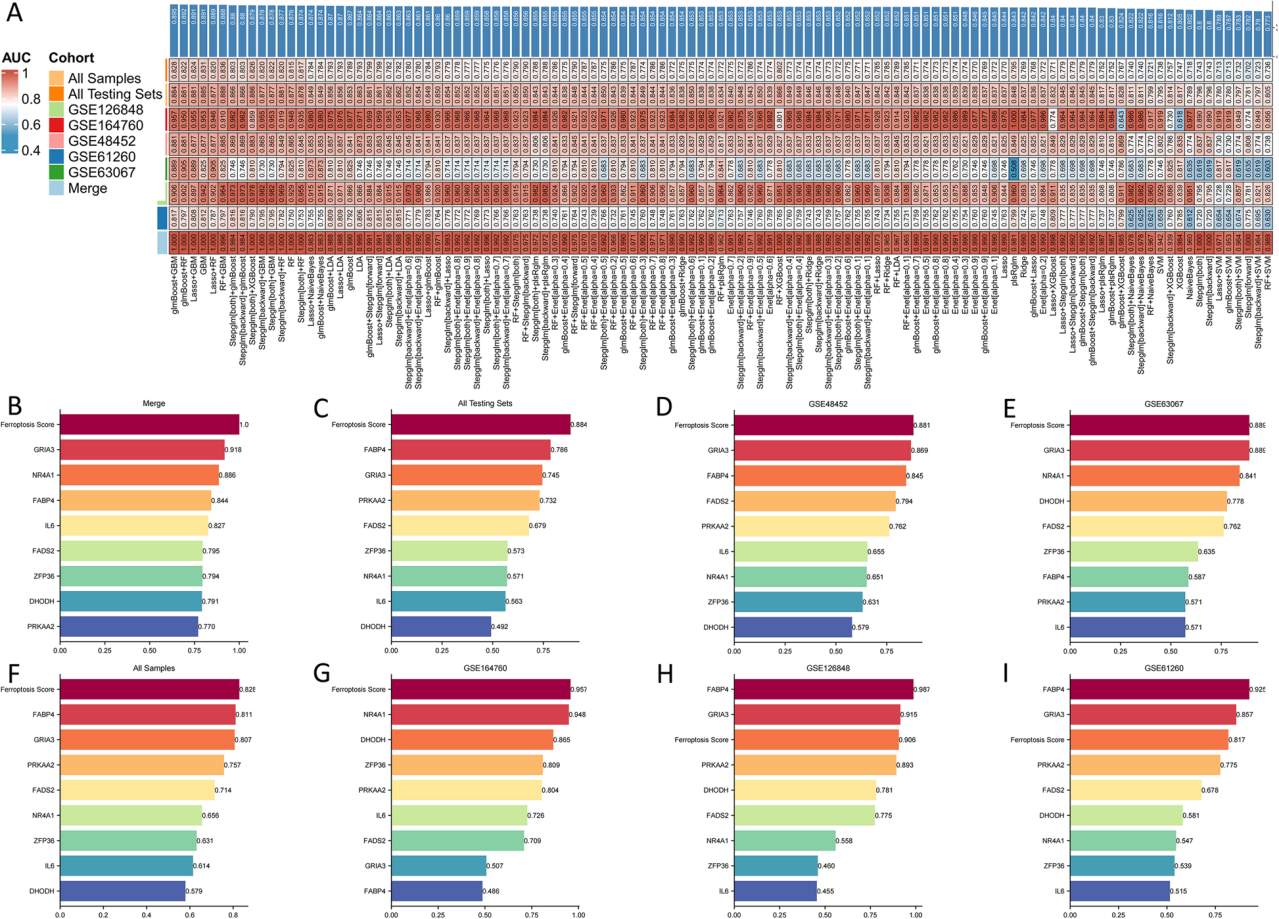
A ferroptosis-related signature (FeRS) of nonalcoholic steatohepatitis (NASH) was developed. **A** The ROC values for diagnosing NASH using the 113 algorithms on the eight sets are shown in a heatmap. **B-I** Based on the eight model genes and FeRS, ROC values have been calculated for eight sets of NASH diagnoses

**Fig. 5:**
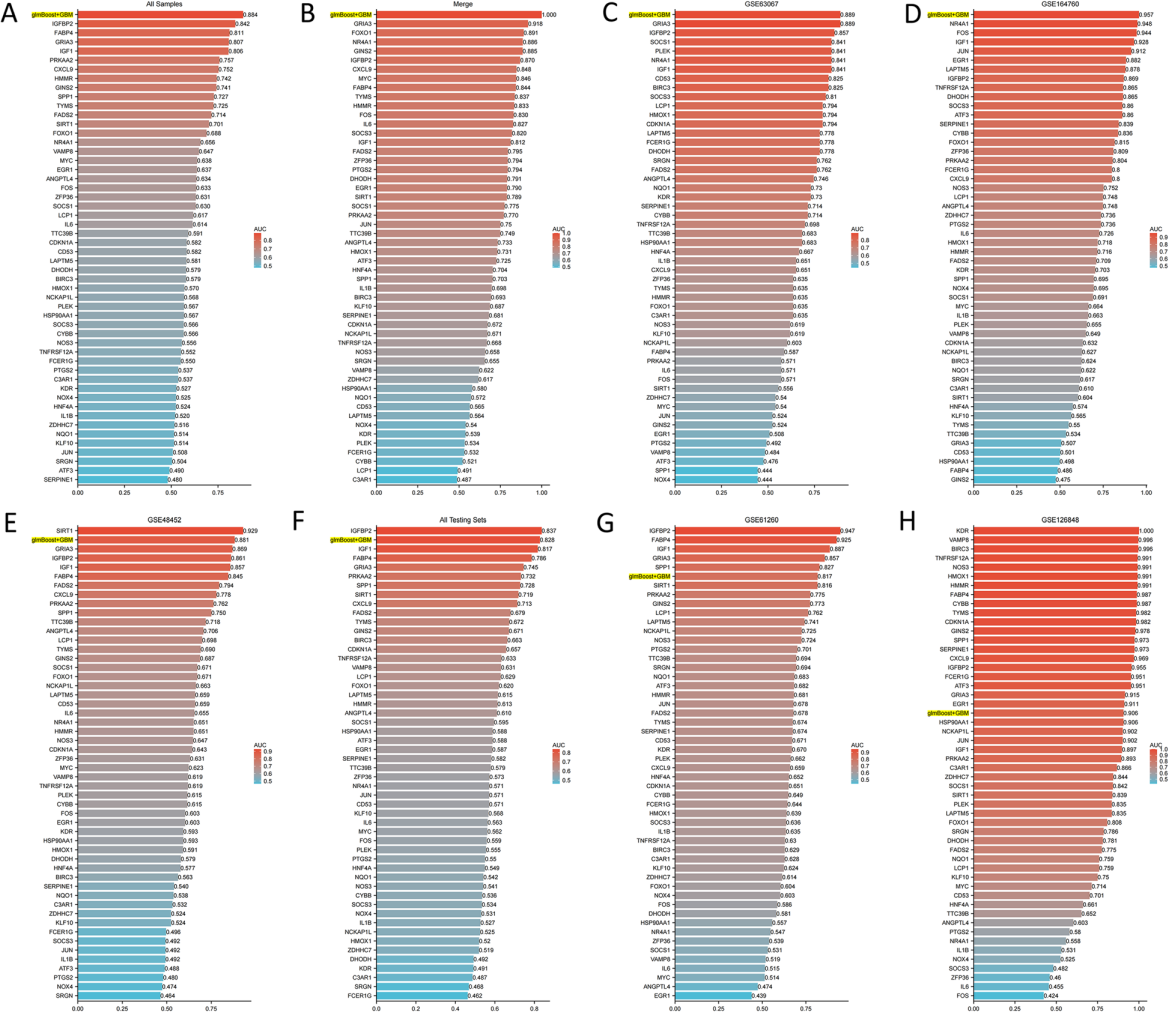
AUC values are shown in bar graphs. **A-H** The ROC values for diagnosing NASH using FeRS and 51 genes across 8 cohorts were calculated and compared

**Fig. 6:**
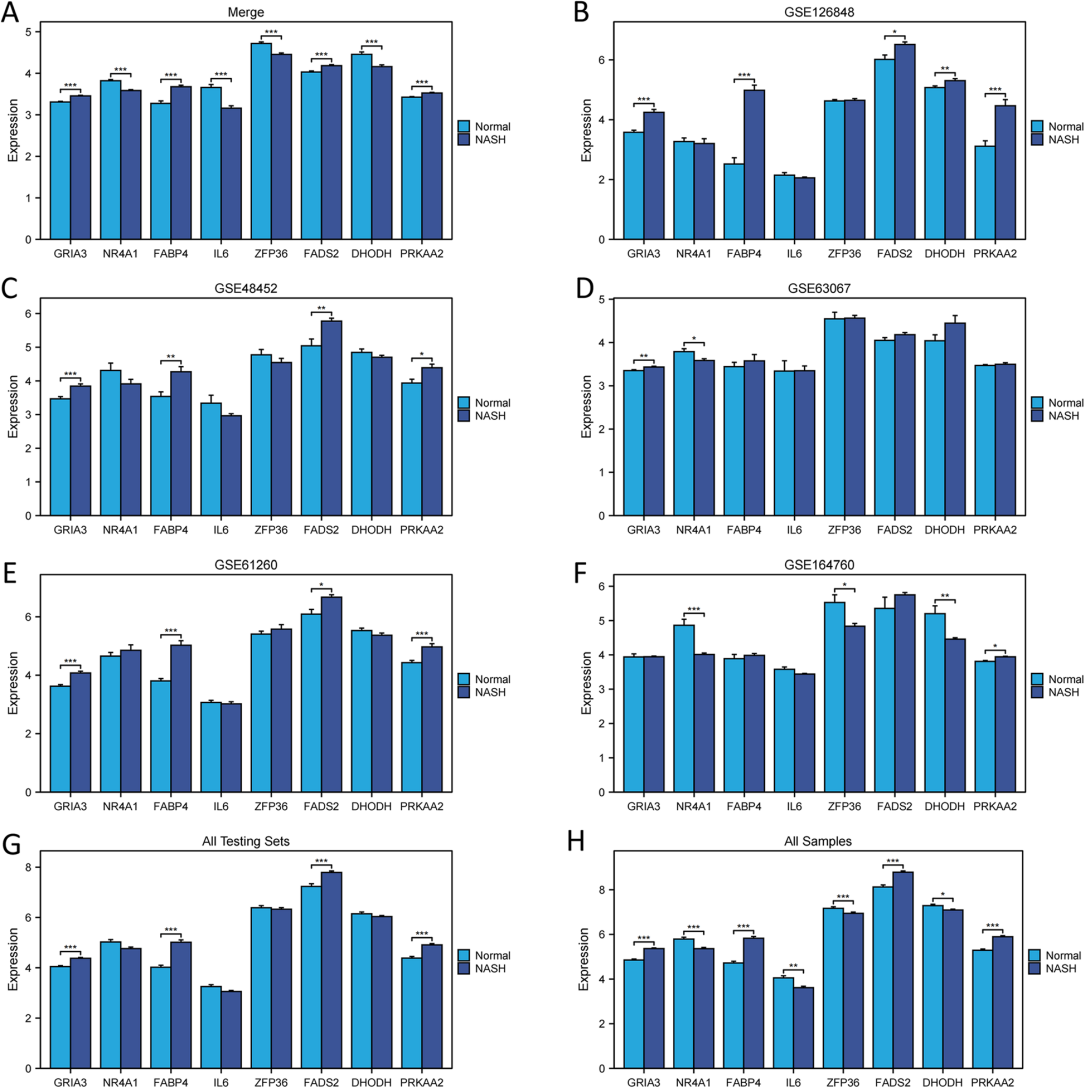
Gene expression histogram differences. **A-H** The 8 model genes were assessed for differences in expression levels. * *P* < 0.05, ** *P* < 0.01, *** *P* < 0.001

After the calculations, “glmBoost+GBM” yielded the best predictive performance among the 113 algorithms tested. It was constructed by applying the “glmBoost+GBM” algorithm to an FeRS consisting of eight FRGs (GRIA3, NR4A1, FABP4, IL6, FADS2, ZFP36, DHODH, and PRKAA2). The ROC values for diagnosing NASH using FeRS in eight sets (Merge cohort, GSE61260 cohort, GSE126848 cohort, GSE63067 cohort, GSE48452 cohort, GSE164760 cohort, All Testing Sets cohort, and All Samples cohort) were 1.000, 0.817, 0.906, 0.889, 0.881, 0.957, 0.884, and 0.828, respectively (Fig. 4A). The model performed well in all seven testing sets, indicating that the “glmBoost+GBM” algorithm has a low risk of overfitting. Additionally, ROC values were plotted to individually diagnose NASH using the eight model genes in eight sets (Fig. 4B-I). Compared to FeRS, these eight model genes did not perform well in each cohort studied. Therefore, the FeRS is more suitable for diagnosing NASH in different cohorts. The model was compared with previously published NASH-related biomarkers. In total, 43 NASH diagnostic genes were identified in nine previous studies [24–32]. Notably, FeRS also exhibited good predictive performance (Fig. 5A-H). The combination of gene expression level differences across the eight cohorts showed that GRIA3, FADS2, FABP4, and PRKAA2 were upregulated in NASH, whereas NR4A1, IL6, ZFP36, and DHODH exhibited the opposite pattern (Fig. 6A-H).

**Fig. 7:**
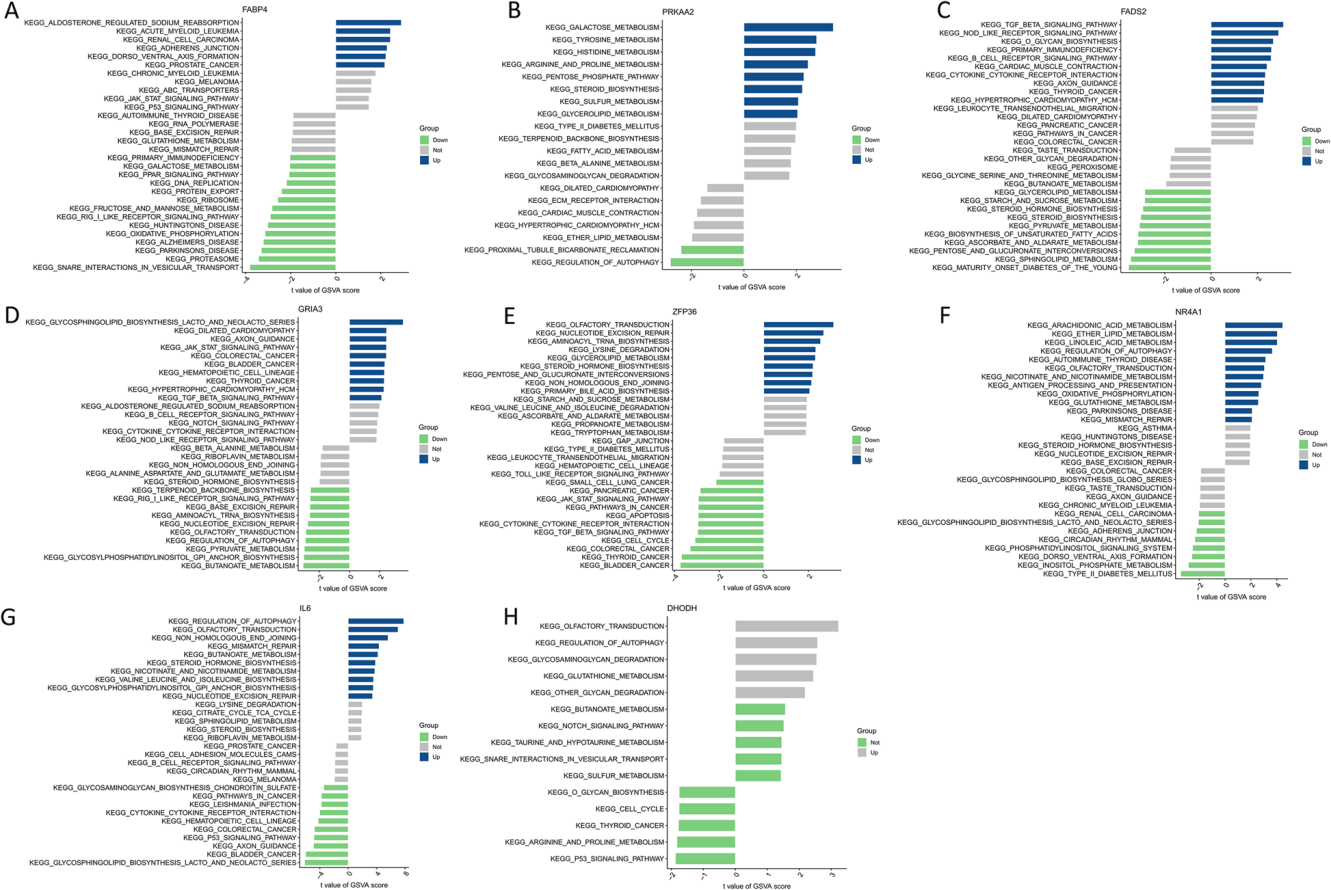
Enrichment pathway analysis. **A-H** The eight model genes were analyzed using GSVA

Notably, model genes were closely associated with specific biological processes. Upregulation of FABP4 was implicated in “adherens junction” and “sodium reabsorption” (Fig. 7A). When PRKAA2 was upregulated, it was significantly enriched in “glycerolipid metabolism” and “steroid biosynthesis.” In addition, it is involved in the metabolism of various amino acids, including arginine, proline, histidine, and tyrosine (Fig. 7B). The upregulation of FADS2 and GRIA3 primarily involves the “TGF-β signaling pathway” associated with the regulation of inflammation (Fig. 7C, D). Furthermore, the upregulation of GRIA3 was significantly enriched in another inflammation-regulating pathway, the “JAK-STAT signaling pathway.” ZFP36 was significantly associated with both inflammation-regulating pathways (Fig. 7E). NR4A1 significantly enriched the pathways associated with “glycosphingolipid biosynthesis,” “circadian rhythms,” and “type 2 diabetes” (Fig. 7F). The downregulation of IL6 primarily enriched the pathways associated with “glycosphingolipid biosynthesis” (Fig. 7G). DHODH downregulation did not significantly enrich any biological pathways (Fig. 7H).

### Enrichment patterns in NASH

**Fig. 8:**
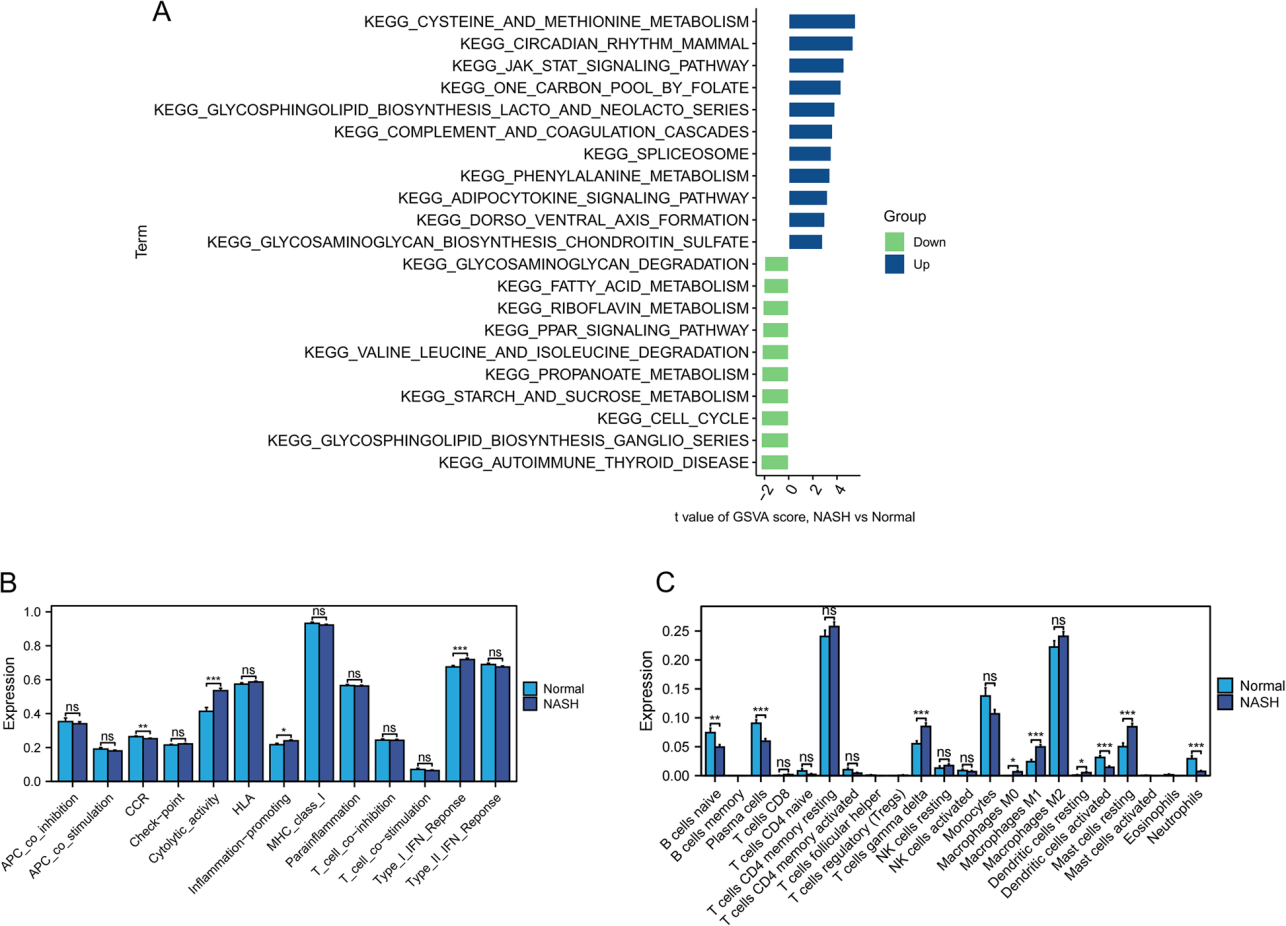
Enrichment patterns of NASH. **A** GSVA enrichment analysis. **B, C** Patterns of immune landscape in patients with NASH. * *P* < 0.05, ** *P* < 0.01, *** *P* < 0.001

A better understanding of the pathogenesis of NASH was obtained by comparing the enriched biological pathways. “Cysteine and methionine metabolism,” “circadian rhythm,” “JAK-STAT signaling pathway,” “phenylalanine metabolism,” and “adipocytokine signaling pathway” exhibited significant upregulation in the NASH group (Fig. 8A).

This study further revealed whether the immune signatures and immune cells affect NASH (Fig. 8B and C). Notably, “type I IFN response,” “inflammation-promoting,” “cytolytic activity,” “T cells gamma delta,” “macrophages M0,” “macrophages M1,” “dendritic cells resting”, and “mast cells resting” were upregulated in NASH, while “CCR,” “B cells naive,” “plasma cells,” “dendritic cells activated,” and “neutrophils” exhibited the opposite trend.

### Enrichment patterns for 8 model genes

**Fig. 9:**
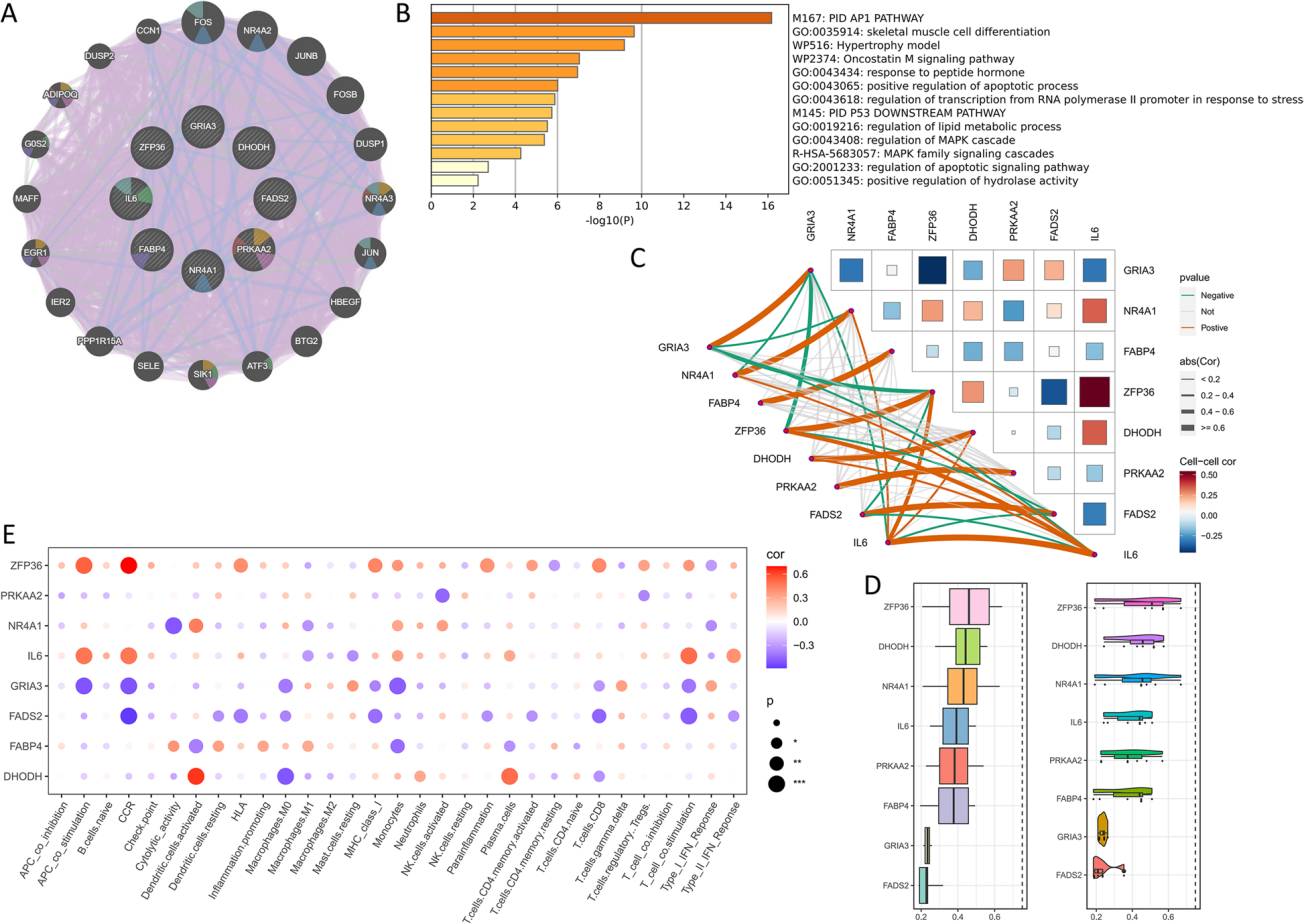
Model gene enrichment patterns. **A** PPI network map. **B** Biological pathway enrichment map. **C** Network map of gene correlation. **D** Friendship analysis. **E** Correlation network map. * *P* < 0.05, ** *P* < 0.01, *** *P* < 0.001

An in-depth exploration of the biological significance of eight model genes was conducted. Twenty genes were closely associated with these eight genes (Fig. 9A). These 20 genes were significantly enriched in “PID AP1 pathway,” “PID P53 downstream pathway,” and “regulation of lipid metabolic processes” (Fig. 9B), as well as in the “MAPK family signaling cascades” associated with inflammation. Subsequently, their interactions were analyzed further in this study (Fig. 9C). ZFP36 ranked as the most important in FeRS, contrasting with FADS2 expression, yet showing synergy with IL6 expression (Fig. 9D). Notably, the downregulation of ZFP36 and IL6 suppressed the immune infiltration status of NASH, whereas the opposite was true for GRIA3 and FADS2 (Fig. 9E).

### Single cell analysis

**Fig. 10:**
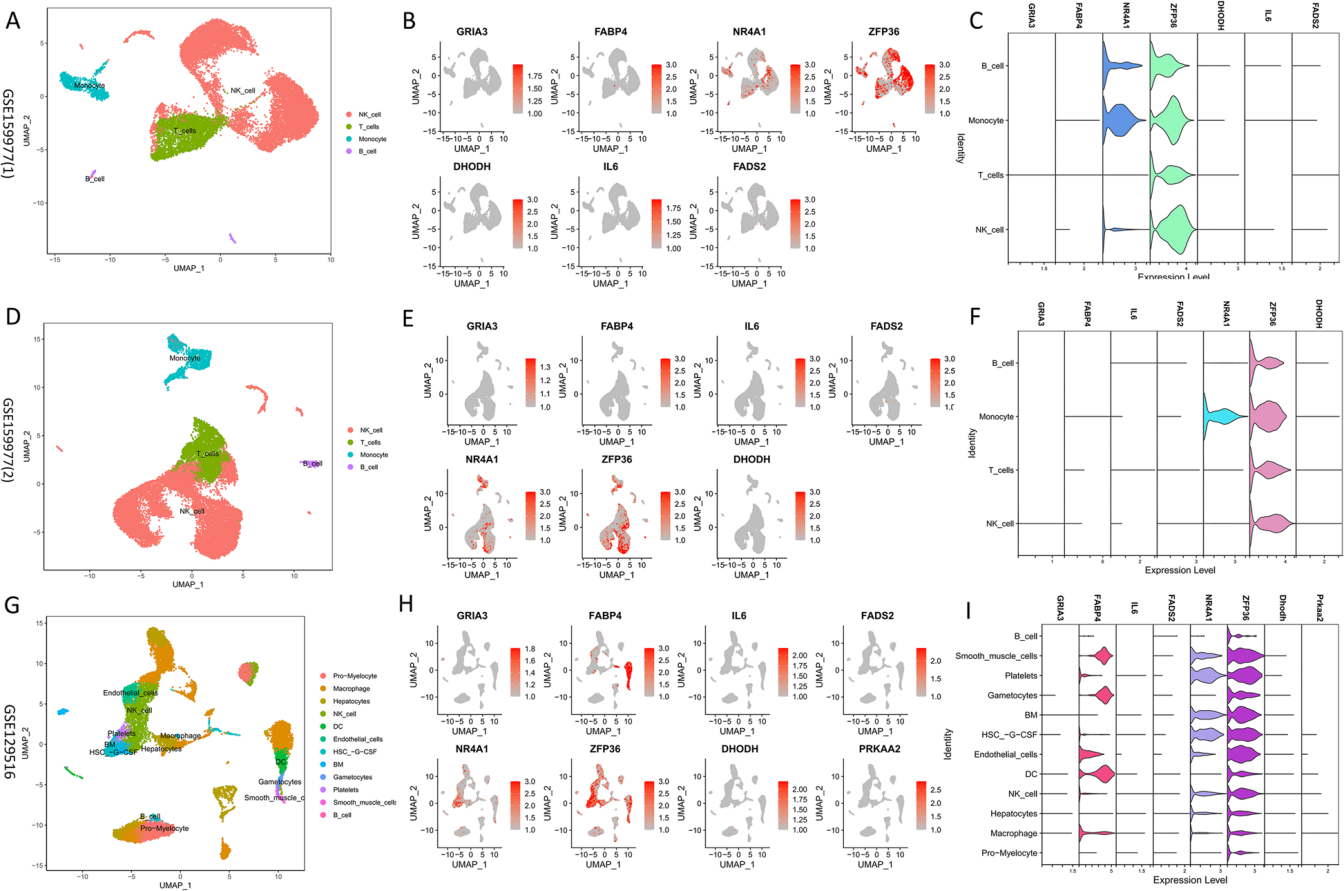
Single-cell annotations. **a** Four immune cells were annotated in GSE159977(1). **b-c** Enrichment landscapes of the model genes in immune cells. **d** Four immune cells were annotated in GSE159977(2).**e-f** Enrichment landscape of the model genes in immune cells. **g** Twelve immune cells were annotated in GSE129516. **h-i** Enrichment landscape of model genes in 12 types of cells

A significant enrichment of these eight model genes was observed in the different single-cell datasets. The GSE159977(1) cohort was annotated using “SingleR” into 4 immune cell types (Fig. 10A). ZFP36 was significantly enriched in all four immune cell types, whereas NR4A1 was enriched only in NK cells, monocytes, and B cells (Fig. 10B and C). The remaining six genes were not significantly enriched. The GSE159977(2) cohort was similarly annotated using “SingleR” (Fig. 10D). By contrast, ZFP36 was significantly enriched in all four of the immune cell types, whereas NR4A1 was enriched only in monocytes (Fig. 10E and F). This suggests that ZFP36 was highly expressed in multiple immune cell types, whereas NR4A1 was primarily upregulated in monocytes. To validate this, we used the mouse single-cell data GSE129516, which was annotated using “SingleR” into 12 cell types (Fig. 10G). ZFP36 was significantly upregulated in B cells, dendritic cells, NK cells, and macrophages (Fig. 10H and I). There was an obvious enrichment of NR4A1 in NK cells and macrophages, whereas FABP4 was markedly enriched in NK cells, dendritic cells, and macrophages.

### Identification of translation regulators

**Fig. 11:**
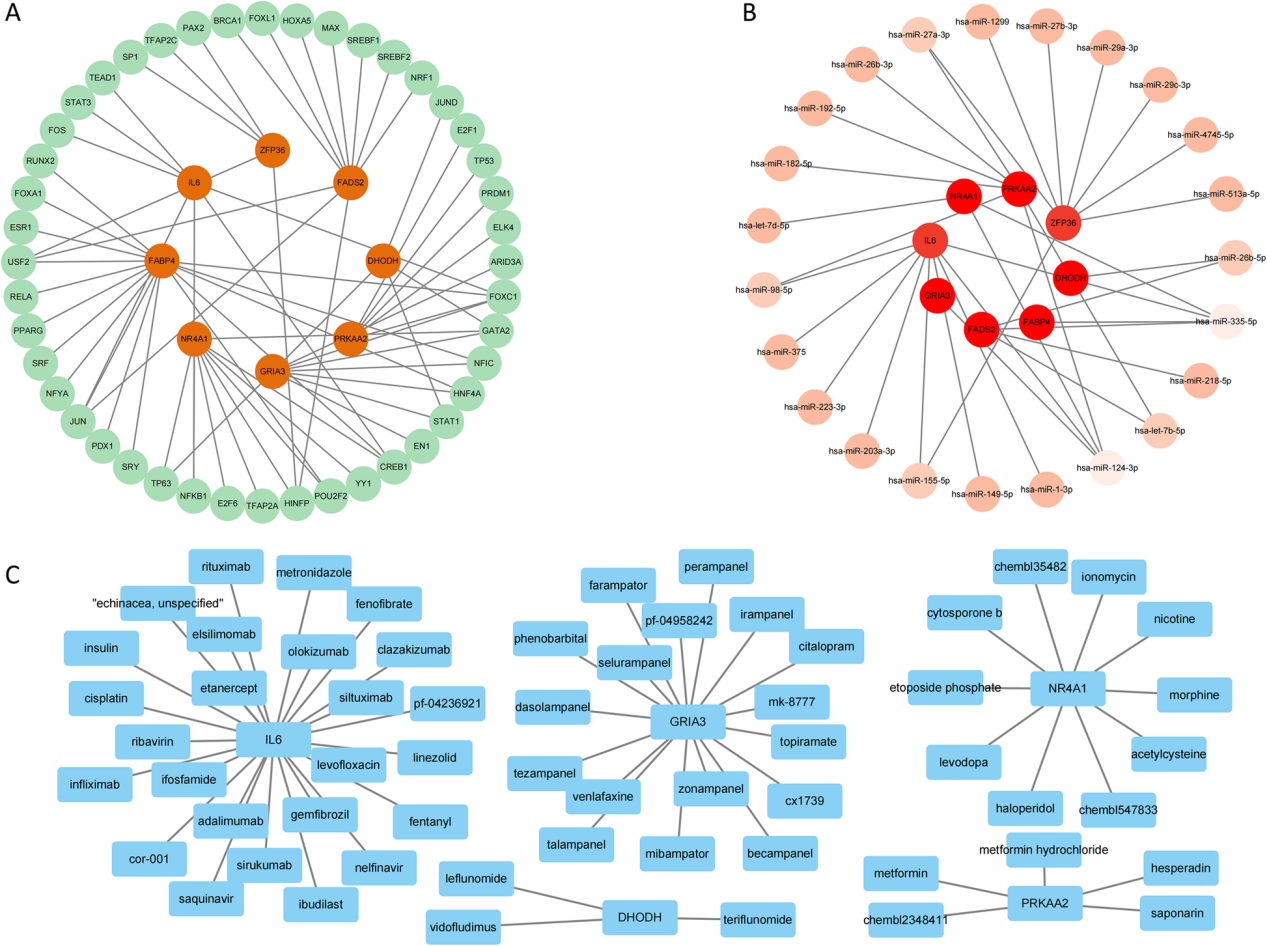
Identification of TFs, miRNAs, and drugs. **a** Forty-four TFs regulate the translation process of the model genes. **b** Twenty-three miRNAs regulate the translation process of the model genes. **c** Predicted 60 therapeutic drugs for NASH

Forty-four TFs and 23 miRNAs were identified (Fig. 11A-B), which likely influenced the development of NASH by regulating the translation of the model genes. In addition, 60 drugs targeting the five model genes were identified (Fig. 11C). This indicates the possibility of curing NASH.

### Patterns of gene expression

**Fig. 12:**
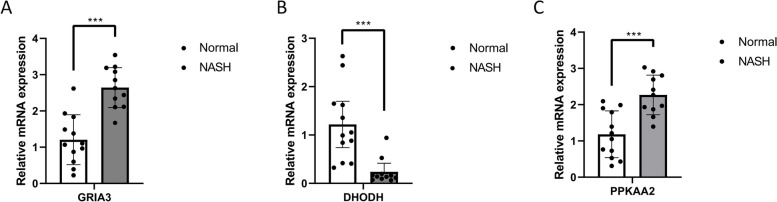
**a-c** GRIA3, DHODH, and PRKAA2 were differentially expressed. *** *P* < 0.001

The genes were validated at the mRNA level using clinical samples. qPCR revealed the upregulation of GRIA3 and PRKAA2 and the downregulation of DHODH in patients with NASH (Fig. 12a–c).

## Discussion

To date, NASH has remained a challenge that has needed to be addressed [33]. Determining the drivers of inflammation is a major problem [34]. Because the liver is a major site for iron ion metabolism, ferroptosis is thought to be involved in the development of NASH [35]. Therefore, studies on ferroptosis may contribute to the management of NASH [36].

The 16 FRGs identified in this study are widely implicated in various metabolic pathways, such as “metabolism of alpha-linolenic acid,” “fat cell differentiation,” “regulation of fat cell differentiation,” and “circadian rhythm genes.” Notably, these genes have been linked to the causes of NASH, such as “fatty liver disease,” “dyslipidemias,” “insulin resistance,” “gestational diabetes,” and “unipolar depression” [37–41]. Sixteen FRGs were processed further. The “glmBoost+GBM” combination algorithm was optimal, which developed a FeRS with 8 genes. This predictive model diagnosed NASH with an ROC value higher than 0.80 in eight datasets. FeRS diagnosed NASH well in all eight datasets compared to other diagnostic markers for NASH, demonstrating the strong generalizability of the model.

A deeper examination of these eight model genes has revealed that ZFP36 ranked first in importance in patients with NASH and was significantly downregulated (P < 0.001). ZFP36 is implicated in the “JAK-STAT signaling pathway” [42] and “TGF-β signaling pathway,” which are closely associated with inflammation regulation [43]. ZFP36 is also associated with immune infiltration in NASH and its expression levels affect most immune signatures and cells. In particular, ZFP36 is positively associated with regulatory T cells and has anti-inflammatory effects. This implies that low ZFP36 expression in patients with NASH may promote the progression of inflammation by suppressing immune infiltration.

TFs can influence gene expression by regulating post-transcriptional translational processes [44]. Five ZFP36 TFs were identified in this study. This discovery facilitates further understanding of the specific mechanism of ZFP36 effects in NASH. Another microRNAs (miRNAs) that post-transcriptionally regulates gene expression was also analyzed [45]. It also inhibits translation and promotes RNA degradation [46]. Eight miRNAs bound to ZFP36 were identified. They may have essential functions in the promotion of NASH progression by ZFP36, affecting both metabolic and inflammatory pathways [47–49].

Additionally, the highly expressed genes GRIA3, FABP4, FADS2, and PRKAA2 have been implicated in lipid metabolism and liver fibrosis. They not only inhibit the anti-inflammatory effects of regulatory T cells but also promote the pro-inflammatory effects of M1 macrophages [50].In contrast, low expression of NR4A1, IL6, and DHODH involves key pathways such as “glycosphingolipid biosynthesis,” “circadian rhythms” and “type 2 diabetes,” activating pro-inflammatory M1 macrophages.

### Study strengths and limitations

The advantage of this study is that compared to liver puncture biopsy, the FeRS not only can be applied conveniently and quickly on a large scale, but also avoids the great risks and pains that invasive operations bring to patients. However, the limitation is that NASH is a heterogeneous disease and the manifestations can vary according to individual differences. The FeRS may not be able to fully capture all potential variants, resulting in limited accuracy for different subtypes of NASH.

## Conclusions

In this study, an FeRS was constructed and eight FRGs were identified. This model provides a noninvasive diagnostic method for NASH. Utilizing this noninvasive model helps to avoid complications associated with liver biopsy. Among the eight genes, ZFP36 ranked first. The downregulation of ZFP36 in NASH is not only involved in the “JAK-STAT pathway” and “TGF-βpathway” associated with inflammation regulation but also inhibits immune infiltration and promotes inflammation progression. The identification of ZFP36 has positive implications for the treatment of NASH, and its activation may inhibit NASH progression.

### Supplementary Information

**Additional file 1.**

**Additional file 2.**

## Data Availability

Raw data were sourced from the GEO (https://www.ncbi.nlm.nih.gov/geo/) and FerrDb V2 (http://www.zhounan.org/ferrdb/current/) databases.
